# SARS-CoV-2 Incubation Period during the Omicron BA.5–Dominant Period in Japan

**DOI:** 10.3201/eid2903.221360

**Published:** 2023-03

**Authors:** Tsuyoshi Ogata, Hideo Tanaka

**Affiliations:** Itako Public Health Center of Ibaraki Prefectural Government, Ibaraki, Japan (T. Ogata);; Public Health Center of Neyagawa City, Osaka, Japan (H. Tanaka)

**Keywords:** COVID-19, Omicron, BA.5, respiratory infections, severe acute respiratory syndrome coronavirus 2, SARS-CoV-2, SARS, coronavirus disease, zoonoses, viruses, coronavirus, incubation period, Japan

## Abstract

The mean virus incubation period during the SARS-CoV-2 Omicron BA.5–dominant period in Japan was 2.6 (95% CI 2.5–2.8) days, which was less than during the Delta-dominant period. Incubation period correlated with shared meals and adult infectors. A shorter incubation suggests a shorter quarantine period for BA.5 than for other variants.

BA.5 is a subvariant of the SARS-CoV-2 Omicron variant (*1*). Since July 2022, Omicron BA.5 has been the dominant variant in Japan (*2*,*3*). According to Japan’s Infectious Diseases Control Law, public health centers (PHCs) must be notified of all COVID-19 cases and conduct contact tracing (*4*). The first patient infected with SARS-CoV-2 Omicron BA.5 was reported on July 4, 2022, to the Itako PHC in Ibaraki Prefecture, Japan. The number of confirmed COVID-19 cases from July 4 through August 19, 2022, was 12,577 in the PHC’s jurisdiction, which has a population of ≈265,000 persons (*5*). In our study, we estimated the incubation period (period between virus exposure and symptom onset) of SARS-CoV-2 Omicron BA.5, determined potential correlations with demographic data and transmission settings, and compared the BA.5 subvariant with other subvariants.

## The Study

We enrolled COVID-19 infector/infectee pairs who lived within the Itako PHC jurisdiction and had a single definite close contact date with patients who had COVID-19 without other potential transmission settings. We calculated the incubation period by using the calendar dates of contact and symptom onset, regardless of duration or number of contacts. If a pair shared meals on July 4 and the infectee had COVID-19 symptoms on July 7, we calculated an incubation period of 3 days. If the pair shared meals on both July 4 and 5, we excluded all data from this pair. Other transmission settings included conversations in the home, a building or car, or outdoors. Procedures used for contact tracing and data collection for patient pairs with COVID-19 were similar to those described previously (*6*,*7*). Persons exposed to SARS-CoV-2 in household, workplace, or school settings were excluded if they might have been exposed at another time. We defined the patient with the later symptom onset in each pair as the infected patient.

We defined patients in the Omicron BA.5–dominant period as those who had symptoms during July 4–August 19, 2022. Genomic sequencing of 528 samples collected in Ibaraki showed 481 (91%) samples were BA.5, 40 were BA.2, and 7 were BA.1 subvariants. We defined patients in the BA.1–dominant period as those who had symptom onset during January 1–February 2, 2022. Of the 1,216 samples collected during January 3–February 6 in Ibaraki, a total of 1,158 (95%) were negative for the SARS-CoV-2 spike protein mutation L452R (*8*). Genomic sequencing showed that 92% of 22,953 variants of concern sampled in Japan during January 3–February 6 were BA.1 (*9*). We defined patients in the Delta-dominant period as those who had symptom onset during July 23–September 14, 2021, and either they or their contacts were confirmed to have L452R-positive SARS-CoV-2. Genomic sequencing detected variants of concern in Japan from July 19–September 13, 2001, as follows: 26,963 cases of B.1.617.2 (Delta), 15,009 cases of B.1.1.7 (Alpha), and 5 cases of other variants (*9*); the L452R mutation was found mostly in the Delta variant.

We extracted data for 266 infector/infectee pairs who had 1 definite date of SARS-CoV-2 exposure as follows: 122 infectees from 108 infectors during the Omicron BA.5–dominant period, 68 infectees from 49 infectors during the BA.1–dominant period, and 76 infectees from 51 infectors during the Delta-dominant period. Patient data during the BA.1–dominant and Delta-dominant periods were obtained from previous studies (*6*,*7*). The mean (+SD) incubation periods were 2.6 (+1.0) days during the BA.5–dominant period, 2.9 (+1.3) days during the BA.1–dominant period, and 3.7 (+1.6) days during the Delta-dominant period.

When we fitted incubation period data from the BA.5–dominant period to parametric distribution models, the Akaike information criterion for gamma distribution was smaller than that for Gaussian, lognormal, and Weibull distribution models. We fitted incubation period data for each subvariant to the gamma distribution model and calculated parameters and 95% CI within a Bayesian inference framework.

The estimated mean incubation period for patients during the BA.5–dominant period was 2.6 (95% CI 2.5–2.8) days, and the median was 2.5 (95% CI 2.3–2.7) days. The mean during the BA.1–dominant period was 2.9 (95% CI 2.6–3.2) days, and the median was 2.7 (95% CI 2.5–3.0) days. During the Delta-dominant period, the mean incubation period was 3.7 (3.4–4.0) days, and the median was 3.5 (3.2–3.8) days. The estimated mean incubation period was shorter for patients during the BA.5–dominant period than during the Delta-dominant period. The 95th percentile distribution for incubation period was estimated at 4.5 (95% CI 4.1–4.9) days during the BA.5–dominant period, 5.2 (95% CI 4.6–5.9) days during the BA.1–dominant period, and 6.1 (95% CI 5.5–6.8) days during the Delta-dominant period (Table 1; Figure).

**Table 1:** Estimated overall mean incubation periods and means within percentiles for Omicron and Delta variants in study of SARS-CoV-2 incubation period during the Omicron BA.5-dominant period in Japan*

Distribution†	BA.5-dominant period, n = 122	BA.1-dominant period, n = 68	Delta-dominant period, n = 76
Overall mean	2.6 (2.5–2.8)	2.9 (2.6–3.2)	3.7 (3.4–4.0)
5th percentile	1.2 (1.1–1.4)	1.2 (1.0–1.5)	1.8 (1.5–2.1)
25th percentile	1.9 (1.8–2.0)	2.0 (1.8–2.3)	2.7 (2.4–3.0)
50th percentile (median)	2.5 (2.3–2.7)	2.7 (2.5–3.0)	3.5 (3.2–3.8)
75th percentile	3.2 (3.0–3.5)	3.6 (3.3–4.0)	4.5 (4.1–4.9)
95th percentile	4.5 (4.1–4.9)	5.2 (4.6–5.9)	6.1 (5.5–6.8)

*Values are mean no. days (95% CI). Means were calculated by fitting to gamma distribution model for SARS-CoV-2 infections during Omicron BA.5–dominant and BA.1–dominant and Delta-dominant periods. †Distribution of patients within incubation period.

**Figure:**
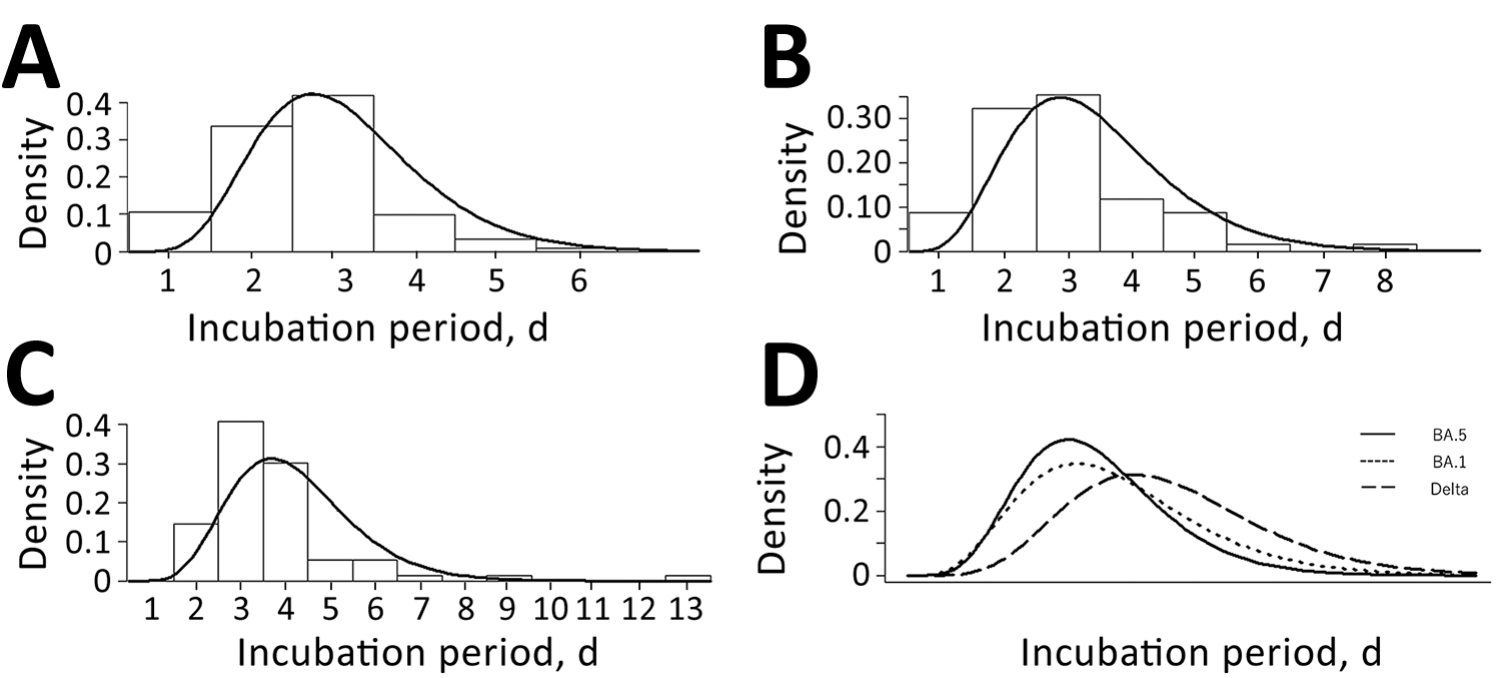
Probability densities for incubation period distribution during different SARS-CoV-2 variant-dominant periods in study of SARS-CoV-2 incubation period during the Omicron BA.5–dominant period in Japan. A–C) Histograms show probability densities for estimated gamma distribution of virus incubation periods for patients during the Omicron BA.5–dominant period (A), Omicron BA.1–dominant period (B), and Delta-dominant period (C). D) Comparison of probability densities for estimated gamma distribution of virus incubation periods among patients during the BA.5-dominant period (solid line), BA.1–dominant period (short-dashed line), and Delta-dominant period (long-dashed line).

We estimated the monovariable mean incubation period of 122 patients during the BA.5–dominant period. The mean incubation period was 2.5 days for patients infected during shared meals, 2.9 days for patients without transmission during shared meals, 3.0 days for patients with infectors who were <19 years of age, and 2.5 days for patients with infectors who were >20 years of age (2.6 days for 79 patients with infectors who were 20–59 years of age and 2.1 days for 16 patients with infectors who were >60 years of age). We performed multivariate gamma regression analyses; transmission during shared meals (p = 0.03) and age of infector (p = 0.007) correlated significantly with incubation period (Table 2).

**Table 2:** Correlations between incubation period and different patient characteristics in study of SARS-CoV-2 incubation period during the Omicron BA.5-dominant period in Japan*

Variables	No. patients	Incubation period, mean no. d (SD)	p value
Total no. patients	122	2.6 (1.0)	NA
Infectee variables			
Sex			0.07
M	55	2.8 (1.0)	NA
F	67	2.5 (1.0)	NA
Age, y			0.13
<19	32	2.8 (1.2)	NA
>20	90	2.6 (0.9)	NA
No. vaccinations			0.66
0–2	54	2.6 (1.0)	NA
3	68	2.6 (1.0)	NA
Transmission—shared meals			0.03
Yes	90	2.5 (0.9)	NA
No	32	2.9 (1.1)	NA
Infector variables			
Sex			0.68
M	68	2.7 (1.0)	NA
F	54	2.5 (1.0)	NA
Age, y			0.007
<19	27	3.0 (1.2)	NA
>20	95	2.5 (0.9)	NA
No. vaccinations			0.83
0–2	59	2.7 (1.0)	NA
3	63	2.6 (0.9)	NA

*We performed multivariable gamma regression analyses of patients during the SARS-CoV-2 Omicron BA.5–dominant period in Japan. NA, not applicable.

## Conclusions

The estimated mean incubation period for patients during the Omicron BA.5–dominant period in this region was 2.6 days. Incubation periods for Omicron BA.1 were reported to be 3.3 days in Norway, 4.6 days in South Korea, 3.2 days in the Netherlands, 3.1 days in Spain, and 2.9 days in Japan (*6*,*10*,*11*); incubation period for BA.2 was 4.4 days in Hong Kong (*12*). 

Reports on incubation periods for BA.5 are sparse. The incubation period in this study for patients during the BA.5–dominant period was markedly shorter than that for patients during the Delta-dominant period; this result is supported by those from previous studies (*10*,*11*). The 95th percentile distribution for incubation period among patients during the BA.5–dominant period appeared shorter than that during the BA.1–dominant period, although the difference was not statistically significant. Our results might warrant a reduction of the quarantine period from 7 to 5 days during the BA.5–dominant period in Japan (*13*).

The incubation period was shorter among patients who had transmission occur during shared meals and those with adult infectors. The incubation period might be influenced by various factors, such as viral load, environmental setting, patient immune response, severity of disease, and selection biases. Infectees exposed while eating do not normally wear masks and may share longer exposure times, so they might be exposed to a higher viral load than those exposed through conversations while wearing a mask. Exposure in restaurants has been shown to increase COVID-19 infection risk (*14*). Furthermore, children usually have mild symptoms, and average viral shedding might be less than that of an adult (*15*).

The first limitation of our study is that the infector and infectee might have been mutually misclassified, leading to overestimation. Second, patient pairs with long incubation periods might be censored during observational periods, and selection bias might result in underestimation. Third, exposures from sources other than the infector of the pair might have been missed. Fourth, the incubation period was calculated by the calendar date and not by real-time intervals. Fifth, the variant type was not confirmed by using genomic sequencing. Finally, increasing patient numbers might have influenced the quality of contact tracing by the PHC.

In summary, our results indicate that the SARS-CoV-2 Omicron BA.5 subvariant has a shorter incubation period than other variants. Shorter incubation time for this variant suggests the quarantine period could be reduced.
